# Genetic diversity of the chemical composition and pod production of *Prosopis juliflora* trees grown in Saudi Arabia

**DOI:** 10.1016/j.heliyon.2023.e21649

**Published:** 2023-11-02

**Authors:** Abdulrahman A. Al-Soqeer, Abdelsalam M. Menshawy, Hassan M. Mousa, Ahmed M. Aggag, Mohamed I. Motawei

**Affiliations:** a The National Research and Development Center for Sustainable Agriculture, Estidamah, Saudi Arabia; b Plant Production and Protection Department, College of Agriculture & Veterinary Medicine, Qassim University, Buraydah 52571, Saudi Arabia; c Food Science and Human Nutrition Department, College of Agriculture & Veterinary Medicine, Qassim University, Saudi Arabia; d Crop Science Department, Faculty of Agriculture, University of Alexandria, Alexandria, Egypt; e Department of Natural Resources and Agricultural Engineering, Faculty of Agriculture, Damanhour University, Damanhour 22511, Egypt

**Keywords:** *Prosopis* plant, Pod production, Chemical composition, Genetic diversity, Cluster analysis, Heatmap

## Abstract

This investigation was conducted to evaluate pod and chemical component traits and the genetic diversity of *Prosopis juliflora* genotypes at two locations. The selected locations were in the middle (Qassim region) and western (Jeddah region) areas, representing two agro-climatic zones of Saudi Arabia. The measured pod characteristics included production, weight, length, filling period, and chemical composition. A wide range of variations in pod yield and chemical traits were observed in the different agro-climatic regions. The results revealed that the mean values ranged from 9.5 kg tree^−1^ (Jeddah) to 14.2 kg tree^−1^ (Qassim) for pod yield, 3.1 g pod^−1^ (Qassim) to 3.7 g pod^−1^ (Jeddah) for pod weight, and 14.8 cm (Qassim) to 16.6 cm (Jiddah) for pod length. The results of genetic diversity indicated that *Prosopis* genotypes in each location were distributed in three different clusters in the two regions at 60 Euclidean distances. The principal component analysis (PCA) showed that the two components (PC1 and PC2) explained 25.03 % and 20.03 % of the overall variance, respectively, which is over 45 % of the variability. The heatmap revealed that genotypes Q20, Q21, and Q24 at the Qassim location and genotypes J1, J6, and J7 at the Jiddah location exhibited positive and significant correlations with pod yield. It can be concluded that *s*uperior *Prosopis* genotypes (Q20, Q21, Q24, J1, J6, and J7) were identified with good traits (pod yield, pod-filling period, and protein %) in each location and may be used in the future for the selection of elite genotypes.

## 1 Introduction

Several tropical developing countries are currently dealing with issues such as malnutrition, desertification, and soil erosion. Most of Saudi Arabia has a very dry climate that is characterized by extreme temperatures, scarce rainfall and soil degradation caused by salinity and low fertility. Recently, the Saudi government has been heavily oriented toward encouraging more projects and ideas that lead to the expansion of the agricultural sector while maintaining water resources [1]. Prosopis juliflora (SW) DC trees are important to the ecology and economy of many arid and semiarid zones where they are found [2]. *P. juliflora* is an essential component of various sustainable land use systems that are improving the livelihoods of rural desert in habitants. It also prevents further soil degradation and assists with land reclamation. It is known for the reversal of desertification and has been suggested as a miracle plant. In addition to its use as a fuel plant, it has varied properties that are useful to humankind [3]. *Prosopis juliflora* DC is a leguminous tree native to arid and semiarid regions of North America, Central America, and the Caribbean, commonly known as “mesquite” [3,4]. The precise date and source of *P. juliflora* introduction to Saudi Arabia are unknown. It was believed to have been introduced several decades ago and is abundant on streets, roadsides, and park plantations; it shows great adaptation.

The potential of *Prosopis juliflora* (mesquite) as a source of food, animal feed, health food, activated charcoal, fuel wood, and a means to stabilize sand dunes and combat desertification was reported [5]. Additionally, it offers a variety of other products and services, as well as cash revenue, which helps to raise the standard of living in rural areas. Mesquite pods have been used for various functions, including usage as human food [[6], [7], [8]], as a source of nutraceuticals [9,10], as a source of energy for raising grill chickens [[10], [11], [12]], and as new medications [11,12] and pesticides, and these functions are greatly impacted by the development of *P. juliflora*for its allelopathy [3]. To the best of our knowledge, there are no data regarding *Prosopis* pod production in Saudi Arabia, and in general, *Prosopis juliflora* starts fruiting at 3–4 years of age*,* and peak pod production occurs at 15–20 years of age [6]. Pod production ranges from a few kilograms to 50–70 kg from tree to tree. Among *Prosopis* species and provenances of the same species, the pod's weight, length, and width also differ [7]. Different studies have reported great variability in pod production from different regions. Harsh and Tewari [8] reported pod production from 5 to 40 kg tree^−1^, depending on rainfall and habitat. For a 5-year-old plantation, the mean pod yield per accession ranged from 7.1 kg tree^−1^ to 0.0 kg tree^−1^ for 25 accessions [9].

*Prosopis* L. is a genus with 44 species belonging to the family Fabaceae and subfamily Mimosoideae. In the dry tropics, of all introduced *Prosopis, P. juliflora* and *P. pallida* perform well, consistently and significantly better than all other species {2}. Several previous studies compared the content of several species of mesquite for chemical composition [[10], [11], [12]]. Ripe pods have a high level of flavor, a modest amount of digestible protein, and a large amount of energy [13]. Proximate analysis of whole *P. juliflora* pods show that they contain 82 %–92 % dry matter, 7 %–16.2 % crude protein, 12.3 %–23.4 % crude fiber, 0.4 %–3.5 % ether extract and 1.4 %–5.8 % ash [14]. *Prosopis* pods are also high in calcium, magnesium, potassium, iron, and zinc and rich in the amino acid lysine [15]. It is extremely challenging to evaluate the selection criteria for multipurpose trees [23]. Due to the extreme diversity in these variables, there are tremendous genetic improvement opportunities in pod production and quality [24]. Sharma et al. [14] investigated the heterogeneity in seed and pod characteristics, performance, and selection of more than 44 *P. juliflora* trees from various locales. Fifteen genotypes were selected from plantations in different regions, and a very high degree of variability was observed among the different provenances due to the obligate out-crossing breeding mechanism of *Prosopis* species [8].

The similarity of any agronomic quantitative characteristics, where the traits are agronomic characteristics of the plant, could be used to quantify genetic links between individuals and populations [25]. Cluster analysis could be used to summarize genetic links among a large number of genotypes and arrange related genotypes into phenotypic groups. Hamza [16] studied genetic diversity within and among *P. juliflora* populations infesting three forests in the Nile River state. Large saline desert areas and land that is severely degraded are present in Saudi Arabia, and there is also a great deal of concern about water resources and their use in agriculture. Inadequate availability of good quality water is one of the major limitations in irrigated agriculture in Saudi Arabia. Therefore, *P. juliflora* may have the potential for cultivation, as it can grow and flourish with a very limited supply of water and tolerate soil and water salinity [1]. Therefore, the objectives of this study were to 1) evaluate *Prosopis juliflora* pod production and chemical components in two regions of Saudi Arabia, 2) quantify genetic diversity within a population of *P. juliflora* and measure differentiation among populations using pod production and chemical components based on Euclidean distance and principal component analysis (PCA), and 3) identify and select *Prosopis* genotypes with superior desirable characteristics that can be domesticated for pod production and quality.

## 2 Materials and methods

### 2.1 Field experimental sites

**Fig. 1:**
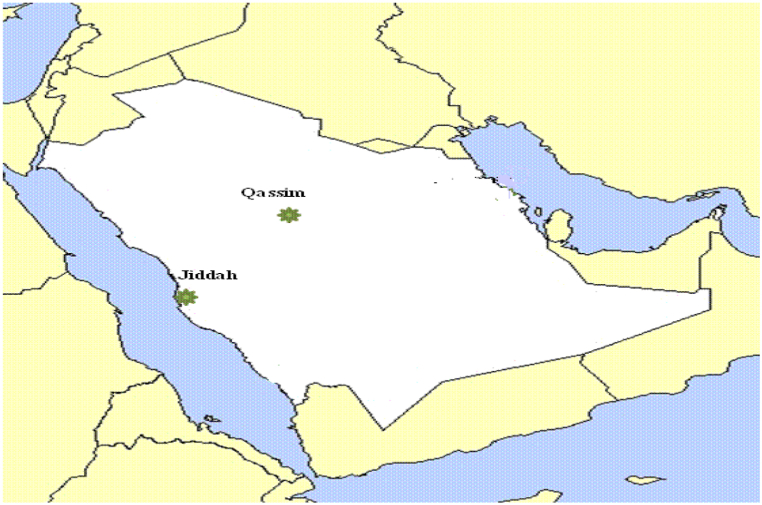
Administrative map of Saudi Arabia showing two selected (Qassim and Jeddah) locations.

This investigation was conducted at the College of Agriculture and Veterinary Medicine, Qassim University, to evaluate pod and chemical component traits for plant populations of *Prosopis* at two sites. The selected sites were in two districts in the middle (Qassim Region) and western (Jeddah Region) areas of Saudi Arabia (Table 1). An administrative map of Saudi Arabia showing the two selected sites under study is presented in Fig. 1. The physical and chemical properties of the soil texture (at 0–25 cm in depth), pH, electrical conductivity (EC), organic matter, total N, available P, and K were analyzed for each site according to Page et al. [17] (Table 2). The climate is typically arid, characterized by exceptionally hot dry summers, subhumid monsoons and cold dry winters. Climatic details recorded at the experimental sites during the period of study are presented in Table 1S.

**Table 1:** Geographic information of the two sites of the sampled populations of *Prosopis juliflora.*

Site name	Symbol	Latitude (N)	Longitude (E)	Elevation (m)	Province
Qassim	QA	26° 18′	43° 46′	646.71	Middle
Jeddah	JE	21° 42′	39° 11′	16.88	Western

**Table 2:** Soil chemical and physical characteristics of the two experimental sites.

Site name	Chemical analysis					
	K	P	N	OM	pH	EC
	––––––––ppm–––––––			%		dS m^−1^
Qassim	34	33.1	15.7	0.40	8.1	5.3
Jeddah	44	10.0	25.0	0.55	7.9	2.4

OM = organic matter; EC = electrical conductivity.

### 2.2 Plant materials

**Fig. 2:**
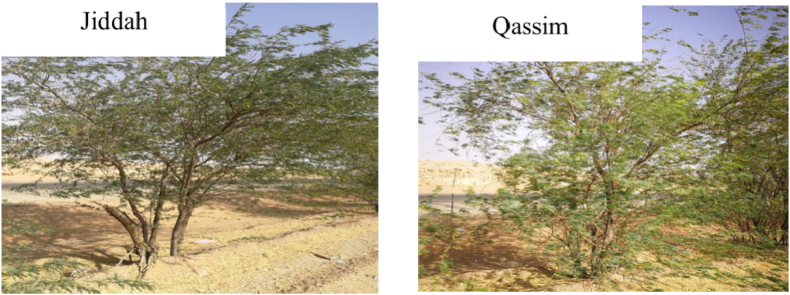
Study sites in Jeddah (left) and Qassim (right).

Sixty *P. juliflora* trees were used in this study. Because mesquite trees are cross-pollinated, each individual tree is considered a genotype according to studies of the genetic diversity of *P. juliflora* by Refs. [1,18]. Thirty genotypes (trees) of similar age were planted in the 2003 season (Fig. 2) with a spacing of 6 × 6 m and collected randomly from each of the middle (Qassim region) and western (Jeddah region) areas of Saudi Arabia to identify the most productive trees. The *Porsopis* trees were collected from Qassim and Jeddah Municipalities, Saudi Arabia and were registered (ENV#1064–09) at King Abdulaziz City for Science and Technology (KACST) (Al-Soqeer et al., 2017).

### 2.3 Field experiments

Groups of *P. juliflora* trees were selected randomly at the two sites. Selected trees in each location were fenced and given numbers (1–30) to compare production and chemical analysis characteristics and study genetic diversity among trees in each location. No fertilizer or irrigation was used during the study at the two locations.

### 2.4 Parameters

#### 2.4.1 Pod production measurements

Mesquite pods were harvested from the previous thirty tagged trees separately for each tree in the two locations by hand at full maturity during May and June 2013. Harvested pods were cleaned, dried at 105 °C for 24 h, weighed, and used for determining pod production tree^−1^ (kg). The weight of the pod was recorded for one hundred pods, and an average was taken for weight pod^−1^ (g) for each tree. Pod length was recorded for each pod, and an average was taken from the 20 pods for each tree. The time of flowering and pod maturity of trees at each location were recorded. The pod-filling period (PFP)is the number of days from the time of flowering to pod maturity (days).

#### 2.4.2 Chemical composition of pods

Chemical analysis of pods for nutrient composition was performed at the Forage Analyses Lab, Department of Animal Production, College of Agriculture and Veterinary Medicine, Qassim University. The dried materials were subjected to chemical analysis according to the AOAC method [19]. Forage quality components were obtained, including crude protein (%), crude fiber (%), sugar (%), ether extract (%) and ash (%). A Foss TECATOR apparatus (model: 2300 Kjeltec) was used to measure the crude protein content, and a Model: Fibertec2010 was used to determine crude fiber content.

### 2.5 Statistical analysis

Basic statistical data, including the mean, range, variance, standard error (SE) and coefficient of variation (CV%), were computed using the Excel Microsoft Computer Program. Pod production and chemical analysis measurements were analyzed using cluster analysis to test for significant differences among the thirty genotypes for each site. A number of phenotypic measures analyzed were found to be resolved into fewer, more comprehensible, and more readily observable dimensions using cluster analysis [30]. To reduce within-cluster sums of squares across all partitions, a hierarchical clustering process employing Ward's minimal variance method was used. The process made use of a technique developed by Hair et al. [32] and detailed by Anderberg [31] that performs a disjoint cluster analysis on the basis of Euclidean distances. Principal component analysis (PCA) and Mahalanobis D2 analysis were used for the estimation of diversity among *Prosopis* genotypes. On the basis of Euclidean distance, the dendrogram was generated. SPSS software was used to perform all of these calculations [33].

Aheatmap (HM) was generated using XLSTAT software version 2019. XLSTAT software was used to classify the data into different clusters having a common trait and was executed to define tree characteristics versus *Prosopis* genotypes groups [20].

## 3 Results

### 3.1 Pod production

Basic statistical data (mean, range, variance, standard error and coefficient of variation) for agronomic traits are presented in Table 3. The overall means of the two locations were first assessed by evaluating all genotypes for each site. The comparisons indicated that the mean values for pod yield varied from 9.5 kg tree^−1^ (Jeddah) to 14.2 kg tree^−1^ (Qassim). Pod weight varied from 3.1 g pod^−1^ (Qassim) to 3.7 g pod^−1^ (Jeddah). The pod length and pod-filling period ranged from 14.8 cm (Qassim) to 16.6 cm (Jeddah) and from 67.8 days (Qassim) to 68.8 days (Jeddah), respectively. The data presented in Table 3 reveal that the pod yield minimum was at Jeddah (0.6 kg tree^−1^) and the maximum was at Qassim (44.1 kg tree^−1^). Minimum and maximum pod weight, pod length and pod-filling period were recorded at Jeddah, 1.0–7.6 g pod^−1^, 6.8–22.7 cm and 47–91 days, respectively. The data in Table 3 revealed that variance estimates for pod yield and pod weight varied from 107.5 at Qassim to 108.1 at Jeddah and from 0.22 at Qassim to 1.63 at Jeddah. The variance values were 4.2–12.6 and 62.4–129.5 for pod length and pod-filling period, respectively. A high value of variance indicates the presence of much variability among trees in each location for each trait, while a small variance indicates the opposite. More promising is the fact that the great variability in the population permitted the possibility for selection of individual trees with outstanding performance. The data in Table 3 show that the SE was 1.9 for pod yield and ranged from 0.09 to 0.23 for pod weight. Moreover, SE values for pod length and pod-filling period varied from 0.4 to 0.6 and from 1.4 to 2.1, respectively. The highest coefficient of variation (CV %) was shown by pod yield, whereas the lowest values were shown by pod-filling period (Table 3).

**Table 3:** Basic statistical data of agronomic traits for 30 *Prosopis juliflora* genotypes for each location in the 2013 season.

	Mean	Range	Variance	SE	CV%
Pod Yield (kg tree^−1^)					
Qassim	14.2a^a^	3.0–44.1	107.5	1.9	73.0
Jeddah	9.5 b	0.6–42.5	108.1	1.9	109.2
Pod weight (g pod^−1^)					
Qassim	3.1 a	2.4–4.2	0.22	0.09	15.1
Jeddah	3.7 a	1.0–7.6	1.63	0.23	34.6
Pod Length (cm)					
Qassim	14.8 a	10.7–18.8	4.2	0.4	13.9
Jeddah	16.6 a	6.8–22.7	12.6	0.6	21.3
Pod-filling period (day)					
Qassim	67.8 a	48–88	62.4	1.4	11.7
Jeddah	68.8 a	47–91	129.5	2.1	16.5

a Means for each factor followed by the same letter are not significantly different at *P < 0.05.*

### 3.2 Chemical composition of pods

Basic statistical data for whole *Prosopis* pods are illustrated in Table 4. The results of the analysis of pods for nutrient composition exhibited a wide range of variation in ash, crude protein, crude fiber, ether extract and sugar contents. The comparisons indicated that the mean values for ash varied from 5.2 % (Jeddah) to 5.7 % (Qassim). Crude protein varied from 12.3 % (Jeddah) to 15.8 % (Qassim). Crude fiber%, ether extract% and sugar % ranged from 12.9 % (Qassim) to 14.3 % (Jeddah), from 1.5 % (Qassim) to 4.4 % (Jeddah) and from 63.7 % (Jeddah) to 64.1 % (Qassim), respectively. The Qassim site had higher values for ash, crude protein and sugar, while it had lower values for ether extract and crude fiber (Table 4). The ranges for characteristics were 4.3–6.8 %, 9.2–18.8 %, 7.5–19.4 %, 0.6–8.9 % and 55.2–72.9 % for ash, crude protein, crude fiber, ether extract and sugar, respectively. The data in Table 4 revealed that variance estimates for ash 0.22 and crude protein ranged from 2.5 in Qassim to 4.3 in Jeddah. The variance values varied from 3.7 to 10.3 %, 0.98–4.5 % and 8.5–19.3 % for crude fiber, ether extract and sugar content, respectively. In general, sugar content recorded the highest values in most cases for variance, followed by crude fiber, while ash content had the lowest variance. The standard error of the mean for the chemical composition of *Prosopis* in the four locations is presented in Table 4. The data showed that the SE values ranged from 0.08 to 0.8 and 0.30–0.40 for ash and crude protein content, respectively. Moreover, SE values for crude fiber, ether extract and sugar content varied from 0.35 to 0.59, from 18 to 39 and from 0.52 to 0.86, respectively. The highest coefficient of variation (CV %) was shown by ether extract, whereas the lowest values were shown by sugar content (Table 4).

**Table 4:** Basic statistical data of chemical analysis traits for 30 *Prosopis juliflora* genotypes for each location in the 2013 season.

	Mean	Range	Variance	SE	CV%
Ash %					
Qassim	5.7 a^a^	4.7–6.8	0.22	0.09	8.2
Jeddah	5.2 a	4.3–6.3	0.22	0.80	8.9
Crude protein %					
Qassim	15.8 a	12.9–18.8	2.5	0.30	10.0
Jeddah	12.3 b	9.2–16.3	4.3	0.40	16.8
Crude fiber %					
Qassim	12.9 a	10.4–17.7	3.7	0.35	14.9
Jeddah	14.3 a	7.5–19.4	10.3	0.59	22.4
Ether extract %					
Qassim	1.5 b	0.6–4.6	0.98	0.18	64.7
Jeddah	4.4 a	0.7–8.9	4.50	0.39	47.9
Sugar %					
Qassim	64.1 a	58.5–68.9	8.5	0.52	4.4
Jeddah	63.7 a	55.2–72.9	19.3	0.80	6.9

a Means for each factor followed by the same letter are not significantly different at *P < 0.05.*

### 3.3 Genetic divergence of Prosopis juliflora genotypes

**Fig. 3:**
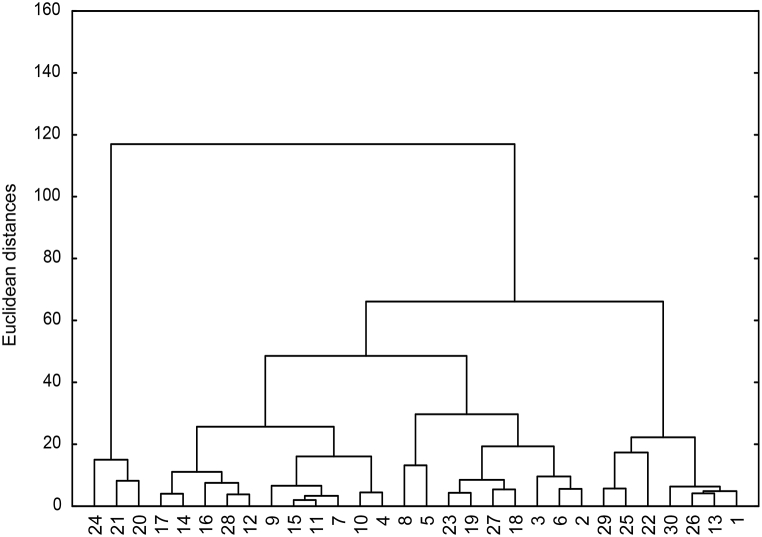
Dendrogram presentation of 30 *Prosopis juliflora* genotypes at the Qassim sampling site.

The genetic divergences based on Euclidean distances using pod and quality characteristics among 30 *Prosopis* genotypes in each location are graphically illustrated as dendrograms and tree diagrams in Fig. 2, Fig. 3. The figures indicate that *Prosopis* genotypes are expected to similarly exhibit a broad spectrum of variabilities and confirm the detected differences among genotypes, as previously mentioned. This conclusion might reflect some sort of dissimilarity between these genotypes for pod production and pod quality.

**Fig. 4:**
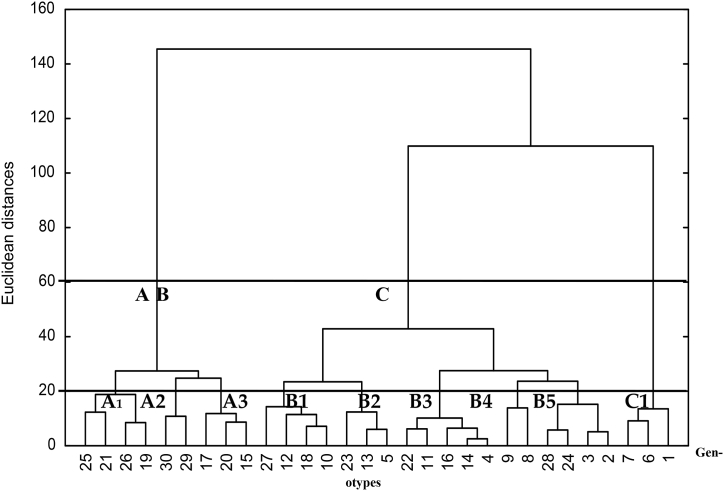
Dendrogram presentation of 30 *Prosopis juliflora* genotypes at the Jeddah location.

The standardized Euclidean distances ranged from 2 to 160 in the two locations (Fig. 3, Fig. 4). The minimum distances were observed between genotypes 11 and 15 (in Qassim) and 4 and 14 (in Jeddah). Meanwhile, the maximum Euclidean distances were observed between genotypes 8 and 21 (in Qassim) and 1 and 20 (in Jeddah).

The clustering pattern of the 30 genotypes was determined at two grouping levels, below 60 and 20 Euclidean distances. The dendrograms of clustering Prosopis genotypes of the two locations using Euclidean distances are illustrated in Fig. 3, Fig. 4.

It is evident from the dendrogram that all 30 genotypes in each location are distributed in three different clusters in the Qassim and Jeddah regions. In the Qassim region, Clusters A, B and C were composed of 3, 20 and 7 genotypes, respectively, but in the Jeddah region, Clusters A, B and C consisted of 9, 18 and 3 genotypes, respectively (Fig. 3, Fig. 4).

The cluster means of the contributing characteristics to the clustering patterns of the two locations are presented in Table 5, Table 6. The data showed that cluster A in the Qassim region was characterized by the highest values for pod yield, pod weight, pod length and sugar %. In contrast, Cluster B had the lowest values for most studied characteristics. In the Jeddah region, Cluster C exhibited the highest values for pod yield, pod length, ash %, crude protein % and EE%, while Cluster A exhibited the lowest values.

**Table 5:** Results by classes derived from PCA.

Class	1	2	3	4
Objects	13	29	6	12
Sum of weights	13	29	6	12
Within-class variance	77.484	86.864	136.461	115.936
Minimum distance to centroid	3.251	4.500	7.700	3.403
Average distance to centroid	8.014	8.541	10.542	9.991
Maximum distance to centroid	13.188	18.033	12.229	13.311
	Q1	Q3	Q20	Q22
	Q2	Q4	Q21	Q25
	Q5	Q7	Q24	Q29
	Q6	Q8	J1	J15
	Q13	Q9	J6	J17
	Q18	Q10	J7	J19
	Q19	Q11		J20
	Q23	Q12		J21
	Q26	Q14		J25
	Q27	Q15		J26
	Q30	Q16		J29
	J8	Q17		J30
	J9	Q28		
		J2		
		J3		
		J4		
		J5		
		J10		
		J11		
		J12		
		J13		
		J14		
		J16		
		J18		
		J22		
		J23		
		J24		
		J27		
		J28

**Table 6:** Agronomic and chemical analysis traits for *Prosopis* genotypes of classes derived from PCA.

Class	Pod Yield (kg tree^−1^)	Pod weight (g pod^−1^)	Pod Length (cm)	Pod-filling period (day)	Ash %	Crud Protein%	Crud Fiber %	EE %	Sugar %
1	16.24 b	3.423 a	15.58 b	66.00 b	5.78 a	14.79 a	12.83 b	2.392 b	64.21 a
2	6.60 c	3.476 a	15.67 b	63.44 b	5.32 b	14.29 a	14.248 a	3.145a	63.01 a
3	38.65 a	3.717 a	17.567 a	66.83 b	5.60 a	14.36 a	13.667 a	3.667a	62.73 a
4	6.45 c	3.083 b	15.208 c	83.25 a	5.35 b	12.56 b	13.042 a	2.883 b	66.15 a

The dendrogram made of the contributed characteristics presented eight clusters of grouping of the 30 genotypes at a similarity lower than 20 Euclidean distances in Qassim. However, genotypes of the Jeddah region were grouped into nine clusters.

In the Qassim region, the eight Clusters A1-C2 were composed of 3, 5, 6, 2, 4, 3, 3 and 4 genotypes, respectively (Fig. 3). The data in Table 5 show that Cluster A1 had the highest pod production and lowest EE%, and the other characteristics were relatively high to moderate. The genotypes in Cluster C1 had the heaviest pods and longest pod-filling period. Cluster C2, composed of genotypes, exhibited the highest values for ash % and crude protein %. The longest pods and highest value of sugar % were obtained with genotypes in Cluster B1. The highest crude fiber % and EE % were obtained with Cluster B5 (Table 5). Regarding the Jeddah location, Clusters A1-C1 consisted of 4, 2, 3, 4, 3, 5, 2, 4 and 3 genotypes, respectively (Fig. 4). The highest mean value for pod production was recorded in Cluster C1 (36.6), followed by Cluster B4 (17.0) (Table 6). Cluster B5 had the genotypes with the highest means of pod length (21.0), crude protein %, and crude fiber %. The highest mean values for pod weight (5.4), ash % (5.7), and EE% (7.0) were recorded for Cluster B4. In terms of pod-filling period and sugar %, Cluster A2 was the best, having the highest mean values (88.5 and 69.1, respectively). Thus, based on a good performance for pod production, Cluster C1, followed by Cluster B4, can be categorized as the best cluster.

### 3.4 Selection and interrelationship of prosopis genotypes with superior desirable characteristics

**Fig. 5:**
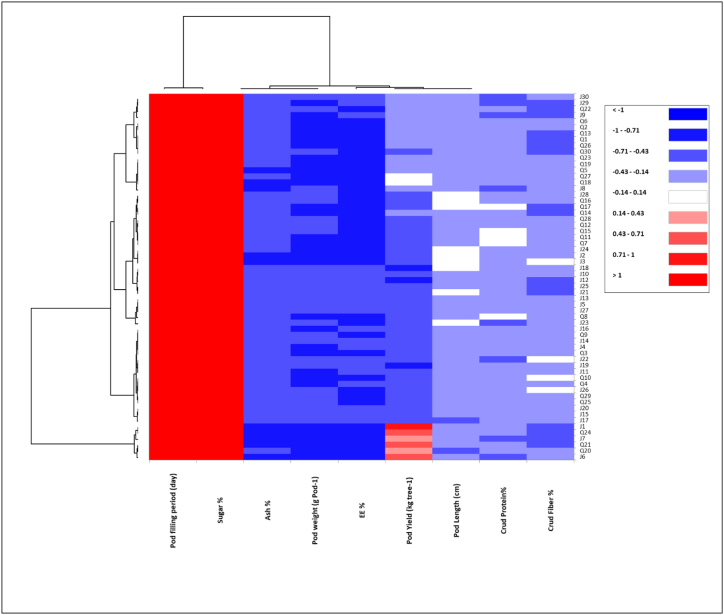
Heatmap (HM) correlation between *Prosopis juliflora* genotypes from the Qassim (Q1–Q30) and Jeddah (J1–J30) locations and evaluated traits.

**Fig. 6:**
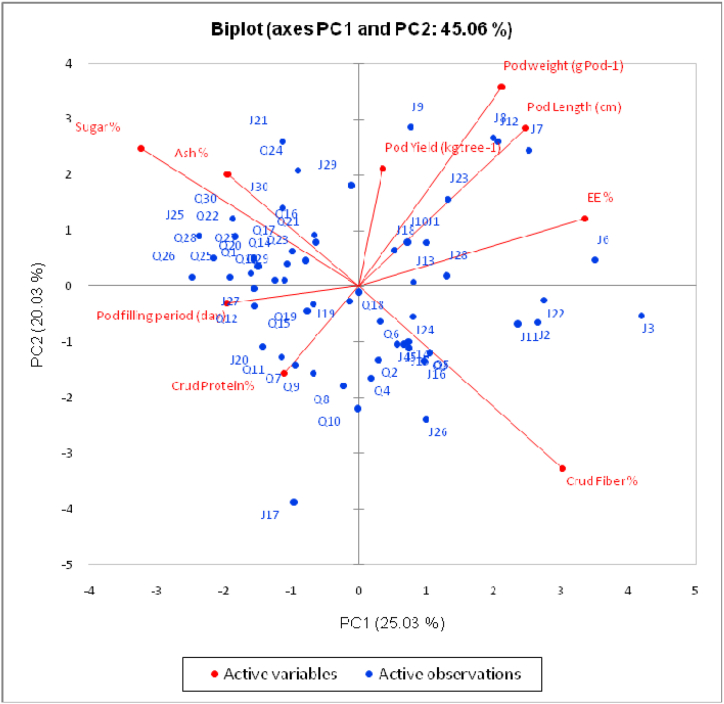
PCA biplot (PC1 vs. PC2) of *Prosopis juliflora* genotypes properties in the Qassim and Jeddah locations.

**Fig. 7:**
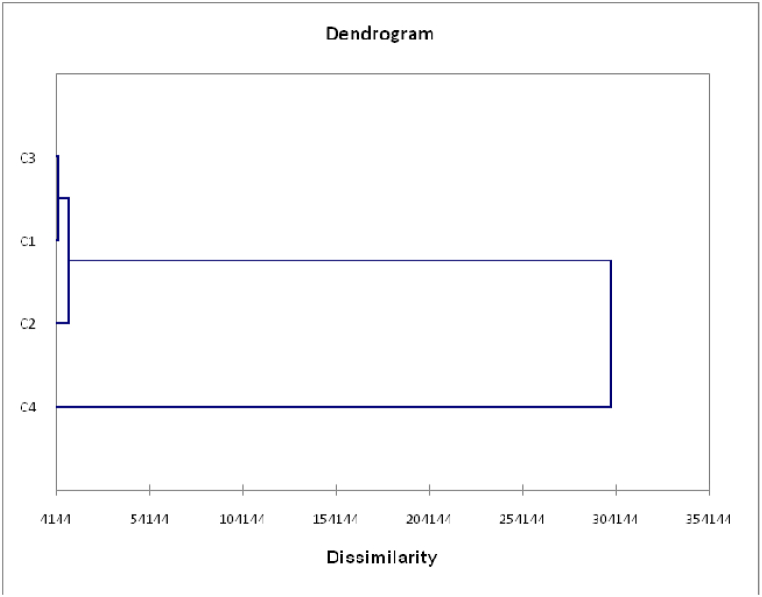
Dendrogram for clustering of *Prosopis juliflora* genotypes.

To explore the relationship between agronomic and chemical composition traits and *Prosopis juliflora* genotypes of both locations, heatmap analysis was employed to disclose this interaction (Fig. 5). The *Prosopis* genotypes of both locations were categorized into four groups (Fig. 5). In addition, principal component analysis (PCA) was performed to highlight the relationship between the studied variables (Fig. 6). Results showed that the two components (PC1 and PC2) explained 25.03 % and 20.03 % of the overall variance, respectively, which is over 45 % of the variability. The PCA biplot revealed that pod yield, pod weight, pod length, pod filling period and crude protein were the major contributor for the PC (Fig. 6). The dendrogram was confirmed by PCA (Fig. 7). *Prosopis juliflora* genotypes were grouped into 4 clusters (Table 5 and Fig. 7). The third group consisted of genotypes Q20, Q21, and Q24 for the Qassim location and genotypes J1, J6, and J7 for the Jeddah location (Table 5). In addition, the third class composed of genotypes exhibited the highest values for pod yield (38.65 kg tree^−1^), pod weight (3.717 g pod^−1^), pod length (17.567 cm), and crude protein % (14.367 %) (Table 6). Interestingly, the heatmap and PCA revealed that genotypes Q20, Q21, and Q24 for the Qassim location and genotypes J1, J6, and J7 for the Jeddah location exhibited positive and significant correlations with pod yield (Fig. 5).

## 4 Discussion

The results of the present study revealed that there is a wide range of variations in pod production of *Prosopis* trees in the two different agro-climatic regions. Pod production was reported to vary from 5 to 40 kg tree^−1^, depending on rainfall and habitat in India (Harsh and Tewari, 1993). Kochare et al. [6] reported that *Prosopis juliflora* started fruiting at 3–4 years of age; 10-year-old plants may yield up to 90 kg pods annually. The pod, its weight, and its length also vary between provenances of *Prosopis juliflora* [7,21]. It is interesting to note that the Jeddah site had a lower pod yield, heavier and longer pods and a longer pod-filling period, while the Qassim site had the reverse trend. A possible explanation for these variations is that the Qassim site had a higher mean precipitation (mm/month) than the Jeddah site. In addition, our results revealed a wide range of variations in the chemical composition traits of *Prosopis* whole pods. The ranges for the chemical analysis traits were 4.3–6., 9.2–18.8, 7.5–19.4, 0.6–8.9 and 55.2–72.9 % for ash, crude protein, crude fiber, ether extract and sugar, respectively, in the present study. These findings are in agreement with many other researchers who found different values of quality characteristics in *Prosopis* genotypes. Shaker et al. [5] reported that the chemical composition of *Prosopis juliflora* pods was 7.37 % ash, 12.83%crude protein, 27.55 % crude fiber and 6.69 % ether extract. Some studies showed that *Prosopis* pods contained 12.7 % CP, 25.4 % CF, 2.6 % EE and 4.8 % ash [6]. These results suggested that it is possible to select genotypes that have high pod production and crude protein content with acceptable levels of the other traits. Generally, these results are in accordance with the findings of many other researchers for pod production and quality traits [22]. Bohra et al. [23] found that the ripened pods of *Prosopis* contain 4.2 % ash, 12.1 % ether extractives, 12.8 % crude protein and 71.0 % total carbohydrates.

The results of minimum and maximum Euclidean distances indicated that the *Prosopis* genotypes of the two locations exhibited different behavior in their genetic divergence. In this regard, Rao et al. [24], using hierarchical clustering by Ward's method for *Jatropha curcas* trees, revealed that trees from different geographic regions were grouped together in a cluster, suggesting that geographical diversity did not match genetic diversity. Additionally, Kumar et al. [25]conducted cluster analysis using Mahalanobis D^2^ analysis among thirty genotypes of bread wheat and stated that all thirty genotypes were grouped into six clusters. Therefore, genetic divergence can provide a visual idea of the variabilities presented in *Prosopis* populations, in addition to ensuring continued genetic improvement [26].

The present study showed that Clusters A1 in Qassim and C1 in Jeddah had the highest mean values for pod production, while they showed high to moderate mean values for the other characteristics. The cluster analysis data could be used to select genetically diverse and superior genotypes from the 30 studied genotypes for each location.

The *Prosopis* genotypes of both locations were categorized into four groups using heatmap (HM) and principal component (PC) analyses. The dendrogram based on pod yield, pod weight, pod length, pod filling period and chemical composition clustered *Prosopis juliflora* genotypes into 4 clusters. The third class composed of genotypes exhibited the highest values for pod yield (38.65 kg tree^−1^), pod weight (3.717 g pod^−1^), pod length (17.567 cm), and crude protein % (14.367 %). Therefore, there is great potential for the development of genotypes for pod traits and quality [27,28]. Additionally, heatmap (HM) analysis revealed that genotypes Q20, Q21, and Q24 for the Qassim location and genotypes J1, J6, and J7 for the Jeddah location exhibited positive and significant correlations with pod yield. Moreover, genotypes Q20 and Q21 were lower in the bioamines histamine and cadaverine than all genotypes in the Qassim location [29]. As reported by Leakey et al. [29], Marula (*Sclerocarya birrea* subsp caffra) cultivar selection would increase productivity and provide an incentive for farmers to plant marula trees. Similar approaches were followed by Sharma et al. [30] in wheat, Cox and Frey [31] in oat and Rao et al. [24] in *Jatropha curcas*.

## 5 Conclusion

A wide range of variations in pod yield and chemical analysis traits were observed in the two different agro-climatic regions (Jeddah and Qassim). The great variability among the genotypes may partially reflect their different genetic backgrounds. Cluster A1 in Qassim and C1 in Jeddah had the highest mean values for pod production, while they showed high to moderate mean values for other characteristics. This study also provides the opportunity to select superior genotypes of *Prosopis* (Q20, Q21, and Q24 for the Qassim location and genotypes J1, J6, and J7) and utilize such genotypes to achieve better and quicker gains. These genotypes exhibited positive and significant correlations with pod yield.

## Author contribution statement

Abdulrahman A. Al-Soqeer: Conceived and designed the experiments; Contributed reagents, materials, analysis tools.

Abdelsalam M. Menshawy: Performed the experiments; Wrote the paper.

Hassan M. Mousa: Contributed reagents, materials, analysis tools or data; Wrote the paper.

Ahmed M. Aggag: Analyzed and interpreted the data; Wrote the paper.

Mohamed I. Motawei: Conceived and designed the experiments; Analyzed and interpreted the data; Wrote the paper.

Data availability statement:

No data was used for the research described in the article.

## Declaration of competing interest

The authors declare that they have no known competing financial interests or personal relationships that could have appeared to influence the work reported in this paper.

## References

[1] Al-Soqeer A.A. (2017). Isolation and identification of allergens and biogenic amines of Prosopis juliflora genotypes. Electron. J. Biotechnol..

[2] Pasiecznik N.M. (2001).

[3] Dave P.N., Bhandari J. (2013). Prosopis julifera: a review. Int. J. Chem. Stud..

[4] Nandagopalan V., A.D a.S.P.A. (2014). Uses and commercial prospects of Prosopis juliflora, in pudukkottai district, south India. Int. J. Phytother..

[5] Shaker Y. (2014). Effect of feeding some salt tolerant fodder shrubs mixture on physiological performance of Shami goats in Southern Sinai, Egypt. J. Am. Sci..

[6] Kochare T., B T., Mengistu A., Goshu G. (2015). The scope of Prosopis juliflora utilization as animal feed in Ethiopia: review. Am. Scient. Res. J. Engin. Technol. Sci..

[7] Sharma N. (1993). Variability and changes in genetic parameters for plant height in prosopis pallida (humboldt and barplant ex. Willdenow) HBK. Ann. Arid Zone.

[8] Harsh L.N., a.J C.T. (1993). Annual Progress Report.

[9] Felker P. (1984). Prosopis pod production—comparison of North American, South American, Hawaiian, and African germplasm in young plantations. Econ. Bot..

[10] Batista A. (2002). In situ ruminal and intestinal nutrient digestibilities of mesquite (Prosopis juliflora) pods. Anim. Feed Sci. Technol..

[11] Freyre M. (2003). Nutritional value of vinal (Prosopis ruscifolia): pods intended for food and feed. Cienc. Tecnol. Aliment..

[12] González Galán A., Corrêa A.D., Piccolo Barcelos M.d.F. (2008). Chemical characterization of integral flour from the Prosopis spp. of Bolivia and Brazil. Arch. Latinoam. Nutr..

[13] Mahgoub O. (2005). Evaluation of Meskit (Prosopis juliflora) pods as a feed for goats. Anim. Feed Sci. Technol..

[14] Choge S. (2007). Prosopis pods as human food, with special reference to Kenya. WaterSA.

[15] Mariam A., Mukhtar A., Mohamed K. (2013). The effect of feeding broiler chicks on prosopis pods flour supplemented with combinations of microbial xylam and phytase enzymes. J. Curr. Res. Sci..

[16] Hamza N.B. (2010). Genetic variation within and among three invasive Prosopis juliflora (Leguminosae) populations in the River Nile State, Sudan. Int. J. Genet. Mol. Biol..

[17] Page A., Miller R., Keeney D. (1982).

[18] Al-Soqeer A.A. (2020). Molecular characterization of mesquite (Prosopis juliflora) genotypes that varied in histamine content using microsatellite markers‏. Biosci. Res..

[19] Horwitz W. (1980).

[20] Xlstat A. (2019).

[21] Saxena S. (1993). Prosopis Species in the Arid and Semi-arid Zone of India. Proceedings of a Conference Held at the Central Arid.

[22] Tewari J. (2011). Desert Environmental Conservation Association (DECO), Jodhpur and Central Arid Zone Research Institute (CAZRI).

[23] Bohra H. (2011).

[24] Rao G. (2008). Genetic associations, variability and diversity in seed characters, growth, reproductive phenology and yield in Jatropha curcas (L.) accessions. Trees (Berl.).

[25] Kumar B., Ruchi G.M.L., Upadhyay A. (2009). Genetic variability, diversity and association of quantitative traits with grain yield in bread wheat (Triticum aestivum L.). Asian J. Agric. Sci..

[26] Martin J., Blake T., Hockett E. (1991). Diversity among North American spring barley cultivars based on coefficients of parentage. Crop Sci..

[27] Leakey R. (2005). Domestication potential of Marula (Sclerocarya birrea subsp. caffra) in South Africa and Namibia: 3. Multiple trait selection. Agrofor. Syst..

[28] Leakey R., Shackleton S., Plessis P.d. (2005). Domestication potential of Marula (Sclerocarya birrea subsp caffra) in South Africa and Namibia: 1. Phenotypic variation in fruit traits. Agrofor. Syst..

[29] Leakey R., Pate K., Lombard C. (2005). Domestication potential of Marula (Sclerocarya birrea subsp caffra) in South Africa and Namibia: 2. Phenotypic variation in nut and kernel traits. Agrofor. Syst..

[30] Sharma P., Gupta P., Balyan H. (1998). Genetic diversity in a large collection of wheats (Triticum spp.). Indian J. Genet. Plant Breed..

[31] Cox T., Frey K. (1985). Complementarity of genes for high groat‐protein percentage from avena sativa L. And A. Sterilis L. 1. Crop Sci..

