# Supplementary material for: Genetic diversity of the chemical composition and pod production of Prosopis juliflora trees grown in Saudi Arabia
Source: Heliyon. 2023 Nov 2;9(11):e21649. doi: 10.1016/j.heliyon.2023.e21649 (PMC10663850; doi:10.1016/j.heliyon.2023.e21649)
Supplement: Multimedia component 1 [file mmc1.doc]

**Table 1S.** Monthly maximum, minimum and average air temperature, mean relative humidity (RH %) and mean of precipitation (mm/month) of two regions during 2013 season*.

| **Month** |  | | **Qassim** | | | | | |  | | Jeddah | | | | |
| --- | --- | --- | --- | --- | --- | --- | --- | --- | --- | --- | --- | --- | --- | --- | --- |
|  | **Temperature °C** | | | |  | RH% | Mean of Precipitation (mm) | Temperature °C | | | | |  | RH% | Mean of Precipitation (mm) |
|  | **Max** | **Min** | | **Average** | |  |  | Max | | Min | | Average | |  |  |
| Jan. | 19.5 | 6.6 | | 13.1 | | 55 | 20.2 | 28.8 | | 18.5 | | 23.7 | | 60 | 12.3 |
| Feb. | 22.5 | 8.4 | | 15.5 | | 44 | 10.8 | 29.5 | | 18.5 | | 24.0 | | 60 | 5.1 |
| Mar | 26.7 | 12.2 | | 19.5 | | 38 | 24.8 | 31.5 | | 19.8 | | 25.7 | | 59 | 1.7 |
| Apr | 32.6 | 17.6 | | 25.1 | | 34 | 24.9 | 34.4 | | 22.2 | | 28.3 | | 56 | 2.3 |
| May | 38.5 | 22.7 | | 30.6 | | 23 | 12.2 | 36.7 | | 24.2 | | 30.5 | | 56 | 0.1 |
| Jun | 42.0 | 24.9 | | 33.5 | | 14 | 0.0 | 37.9 | | 25.0 | | 31.5 | | 57 | 0.0 |
| Jul | 43.1 | 25.9 | | 34.5 | | 13 | 0.1 | 39.0 | | 26.6 | | 32.8 | | 53 | 0.4 |
| Aug | 43.2 | 26.1 | | 34.7 | | 14 | 0.0 | 38.3 | | 27.4 | | 32.9 | | 59 | 0.3 |
| Sep | 40.8 | 23.3 | | 32.1 | | 15 | 0.1 | 37.1 | | 26.4 | | 31.8 | | 66 | 0.1 |
| Oct | 35.2 | 18.5 | | 26.9 | | 24 | 3.6 | 36.2 | | 23.9 | | 30.1 | | 66 | 0.8 |
| Nov | 26.8 | 12.9 | | 19.9 | | 44 | 23.2 | 33.1 | | 22.2 | | 27.7 | | 63 | 18.9 |
| Dec | 21.4 | 8.1 | | 14.8 | | 54 | 13.9 | 30.4 | | 20.0 | | 25.2 | | 61 | 10 |

* Source, according to the Presidency of Meteorology & Environmental.

Table 2 S: Agronomic and chemical analysis traits for 30 prosopis genotypes at Qassim location, 2013 season.

| Tree # | Pod Yield (kg tree^-1^) | Pod weight (g Pod^-1^) | Pod Length (cm) | Pod filling period (day) | Ash % | Crud Protein% | Crud Fiber % | EE % | Sugar % |
| --- | --- | --- | --- | --- | --- | --- | --- | --- | --- |
| 1 | 13.5 | 3.0 | 15.8 | 70 | 6.5 | 18.3 | 11.1 | 1.3 | 62.8 |
| 2 | 18.0 | 2.4 | 13.6 | 65 | 5.4 | 15.1 | 15.5 | 3.3 | 60.7 |
| 3 | 9.6 | 3.0 | 15.2 | 65 | 5.4 | 16.2 | 15.2 | 4.6 | 58.5 |
| 4 | 6.0 | 2.7 | 13.5 | 71 | 5.3 | 16.9 | 14.4 | 3.9 | 59.5 |
| 5 | 16.5 | 3.2 | 14.5 | 54 | 5.1 | 16.2 | 15.7 | 2.9 | 60.0 |
| 6 | 16.5 | 2.9 | 17.8 | 67 | 6.0 | 16.9 | 15.6 | 2.0 | 59.5 |
| 7 | 4.5 | 2.4 | 13.4 | 64 | 5.8 | 17.3 | 13.6 | 1.5 | 61.8 |
| 8 | 6.0 | 2.6 | 10.7 | 48 | 5.0 | 16.3 | 13.8 | 2.1 | 62.9 |
| 9 | 3.0 | 2.8 | 11.3 | 66 | 5.4 | 15.0 | 14.6 | 2.0 | 63.1 |
| 10 | 5.1 | 2.5 | 12.6 | 72 | 5.8 | 15.2 | 17.7 | 2.1 | 59.3 |
| 11 | 6.9 | 2.6 | 13.3 | 65 | 5.4 | 17.7 | 12.7 | 1.0 | 63.2 |
| 12 | 6.6 | 2.9 | 13.2 | 64 | 5.8 | 14.7 | 12.5 | 0.7 | 66.3 |
| 13 | 15.0 | 3.3 | 15.7 | 73 | 5.4 | 17.3 | 11.0 | 1.3 | 65.0 |
| 14 | 12.0 | 3.3 | 17.2 | 65 | 5.4 | 16.9 | 10.8 | 1.0 | 66.0 |
| 15 | 6.0 | 3.7 | 14.7 | 65 | 5.7 | 17.7 | 12.8 | 1.0 | 62.9 |
| 16 | 9.6 | 3.6 | 18.8 | 64 | 5.1 | 13.5 | 11.8 | 0.7 | 68.9 |
| 17 | 10.8 | 3.3 | 18.7 | 64 | 6.1 | 18.8 | 10.4 | 1.4 | 63.3 |
| 18 | 21.0 | 2.9 | 16.5 | 62 | 5.4 | 14.2 | 14.4 | 1.8 | 64.2 |
| 19 | 16.8 | 3.3 | 15.1 | 66 | 4.7 | 15.4 | 12.8 | 0.6 | 66.5 |
| 20 | 36.0 | 3.1 | 12.4 | 76 | 5.9 | 13.6 | 13.7 | 0.9 | 66.0 |
| 21 | 42.0 | 3.4 | 17.1 | 78 | 5.4 | 15.5 | 13.4 | 0.9 | 64.8 |
| 22 | 18.0 | 4.2 | 15.2 | 88 | 5.8 | 16.0 | 11.2 | 0.7 | 66.3 |
| 23 | 16.5 | 3.7 | 14.4 | 63 | 5.8 | 12.9 | 13.9 | 0.8 | 66.7 |
| 24 | 44.1 | 3.9 | 17.7 | 66 | 6.0 | 14.9 | 11.7 | 0.7 | 66.7 |
| 25 | 6.0 | 3.0 | 15.3 | 78 | 6.0 | 16.5 | 10.6 | 1.5 | 65.5 |
| 26 | 12.0 | 2.7 | 13.7 | 74 | 6.8 | 17.5 | 10.6 | 1.0 | 64.1 |
| 27 | 21.0 | 2.7 | 13.7 | 60 | 6.0 | 13.8 | 11.7 | 1.2 | 67.3 |
| 28 | 9.0 | 3.0 | 13.1 | 65 | 6.1 | 14.2 | 10.5 | 1.0 | 68.2 |
| 29 | 7.5 | 3.8 | 14.7 | 81 | 5.8 | 13.4 | 13.9 | 1.2 | 65.7 |
| 30 | 10.5 | 3.6 | 16.6 | 74 | 6.5 | 15.3 | 10.7 | 1.0 | 66.6 |

Table 3 S: Agronomic and chemical analysis traits for 30 prosopis genotypes at Jiddahlocation, 2013 season.

| Tree # | Pod Yield (kg tree^-1^) | Pod weight (g Pod^-1^) | Pod Length (cm) | Pod filling period (day) | Ash % | Crud Protein % | Crud Fiber % | EE % | Sugar % |
| --- | --- | --- | --- | --- | --- | --- | --- | --- | --- |
| 1 | 42.5 | 3.2 | 18.5 | 59 | 5.0 | 16.3 | 12.7 | 3.8 | 62.2 |
| 2 | 11.4 | 3.9 | 20.3 | 61 | 5.7 | 16.4 | 18.1 | 4.4 | 55.5 |
| 3 | 9.6 | 4.7 | 22.7 | 59 | 4.3 | 14.0 | 19.4 | 4.9 | 57.3 |
| 4 | 7.9 | 3.2 | 13.1 | 68 | 5.0 | 14.1 | 14.5 | 4.5 | 61.9 |
| 5 | 2.5 | 2.5 | 14.6 | 58 | 4.9 | 10.9 | 15.6 | 3.7 | 64.9 |
| 6 | 35.2 | 3.6 | 19.4 | 63 | 5.6 | 13.7 | 16.8 | 8.8 | 55.2 |
| 7 | 32.1 | 5.1 | 20.3 | 59 | 5.7 | 12.2 | 13.7 | 6.9 | 61.5 |
| 8 | 15.8 | 6.2 | 18.7 | 59 | 5.3 | 9.2 | 13.9 | 5.0 | 66.5 |
| 9 | 18.1 | 4.6 | 16.5 | 71 | 6.3 | 10.2 | 9.9 | 8.9 | 64.8 |
| 10 | 5.3 | 3.6 | 16.0 | 63 | 5.4 | 12.3 | 11.4 | 6.6 | 64.3 |
| 11 | 8.7 | 3.2 | 16.5 | 71 | 5.0 | 11.9 | 16.2 | 7.9 | 59.1 |
| 12 | 2.2 | 7.6 | 15.8 | 60 | 5.2 | 16.3 | 8.7 | 8.2 | 61.7 |
| 13 | 4.0 | 3.2 | 15.2 | 53 | 5.0 | 11.3 | 13.1 | 5.0 | 65.6 |
| 14 | 5.8 | 3.5 | 13.4 | 68 | 5.3 | 13.8 | 15.6 | 4.2 | 61.2 |
| 15 | 4.4 | 3.0 | 16.7 | 90 | 5.3 | 12.3 | 17.2 | 4.3 | 60.9 |
| 16 | 3.6 | 3.0 | 15.5 | 65 | 5.0 | 13.3 | 16.7 | 3.2 | 61.8 |
| 17 | 0.8 | 1.0 | 6.8 | 85 | 4.5 | 12.7 | 17.0 | 3.2 | 62.6 |
| 18 | 2.3 | 4.0 | 18.7 | 66 | 5.1 | 11.8 | 13.8 | 2.8 | 66.5 |
| 19 | 0.6 | 3.2 | 15.9 | 76 | 5.5 | 11.4 | 14.9 | 3.0 | 65.2 |
| 20 | 1.3 | 1.5 | 13.8 | 89 | 5.4 | 10.0 | 14.3 | 3.1 | 67.1 |
| 21 | 7.7 | 3.2 | 21.0 | 76 | 6.0 | 12.3 | 7.5 | 4.8 | 69.4 |
| 22 | 6.0 | 5.0 | 16.7 | 68 | 5.1 | 10.7 | 19.1 | 5.1 | 60.1 |
| 23 | 5.5 | 4.5 | 19.7 | 47 | 5.9 | 9.5 | 15.4 | 2.8 | 66.4 |
| 24 | 10.2 | 2.5 | 20.9 | 67 | 5.7 | 14.5 | 16.6 | 2.1 | 61.2 |
| 25 | 1.4 | 3.5 | 12.4 | 80 | 4.9 | 11.4 | 8.3 | 2.4 | 72.9 |
| 26 | 2.5 | 3.1 | 15.7 | 79 | 4.5 | 15.3 | 18.4 | 1.9 | 59.8 |
| 27 | 2.0 | 3.5 | 9.5 | 64 | 4.8 | 10.5 | 11.2 | 2.1 | 71.3 |
| 28 | 9.3 | 4.5 | 20.2 | 64 | 4.8 | 12.7 | 17.0 | 0.7 | 64.8 |
| 29 | 16.7 | 3.0 | 20.1 | 86 | 5.7 | 9.8 | 11.6 | 5.7 | 67.2 |
| 30 | 10.6 | 4.5 | 14.9 | 91 | 4.9 | 9.7 | 11.6 | 2.8 | 71.2 |
